# Timepix3: Compensation of Thermal Distortion of Energy Measurement

**DOI:** 10.3390/s23063362

**Published:** 2023-03-22

**Authors:** Martin Urban, Ondrej Nentvich, Lukas Marek, David Hladik, Rene Hudec, Ladislav Sieger

**Affiliations:** 1 Faculty of Electrical Engineering, Czech Technical University in Prague, Technicka 2, 166 27 Prague 6, Czech Republic; 2 Faculty of Mathematics and Physics, Charles University, V Holesovickach 2, 180 00 Prague 8, Czech Republic; 3 Advacam, s.r.o., U Pergamenky 1145/12, 170 00 Prague 7, Czech Republic

**Keywords:** Timepix3, X-ray detector, energy measurement, temperature effects, compensations

## Abstract

The Timepix3 is a hybrid pixellated radiation detector consisting of a 256 px × 256 px radiation-sensitive matrix. Research has shown that it is susceptible to energy spectrum distortion due to temperature variations. This can lead to a relative measurement error of up to 35% in the tested temperature range of 10 °C to 70 °C. To overcome this issue, this study proposes a complex compensation method to reduce the error to less than 1%. The compensation method was tested with different radiation sources, focusing on energy peaks up to 100 keV. The results of the study showed that a general model for temperature distortion compensation could be established, where the error in the X-ray fluorescence spectrum of Lead (74.97 keV) was reduced from 22% to less than 2% for 60 °C after the correction was applied. The validity of the model was also verified at temperatures below 0 °C, where the relative measurement error for the Tin peak (25.27 keV) was reduced from 11.4% to 2.1% at −40 °C. The results of this study demonstrate the effectiveness of the proposed compensation method and models in significantly improving the accuracy of energy measurements. This has implications for various fields of research and industry that require accurate radiation energy measurements and cannot afford to use power for cooling or temperature stabilisation of the detector.

## 1. Introduction

Timepix3 (TPX3) is a hybrid single photon counting pixel detector with semiconductor sensors. Due to its hybrid structure, which allows various combinations of thickness and material types of the semiconducting sensor with the same electronics of the readout chip, in dependence on the desired application, it is a handy, popular, and widespread detector in several different scientific and industrial fields [1]. Typical applications of this type of detector include usage in medicine [2,3], art [4], quality control and material properties, as well as neutron detection [5,6,7] or radiation source localisation and monitoring of the radiation environment [8,9]. Timepix detectors are also frequently used in the scientific domain, such as the ATLAS experiment [10], suborbital rockets [11,12], and applications in space [13], either on board the International Space Station [14,15] or as part of a separate satellite [16,17,18] or CubeSat mission [19,20,21,22,23,24] to monitor space weather, solar flares [25,26] or other scientifically significant radiation phenomena.

Furthermore, the novel X-ray and high-energy space experiments focusing on X-rays require suitable focal detectors to record the images provided by the X-ray optics of these devices. Albeit there are several possibilities, the pixel detectors play an important and increasing role because they meet the requirements to be used on miniature satellites such as CubeSats: they are small, lightweight, energy-efficient, and less expensive than other options. However, such an application requires the detector’s working temperature range to be broad, −20 °C to 60 °C.

Due to the widespread use of detectors with the TPX3 chip, it can be challenging to maintain all the standard environmental conditions for measurements. Namely, the operating temperature of the detector. The TPX3 with Silicon detection layer has been shown to be highly dependent on the operating temperature [27,28].

In situations where it is not possible or economically realistic to stabilise the operating temperature of the detector, it is necessary to compensate for its influence. This article deals with the possibilities and methodology of how to approach this compensation and the evaluation of the effectiveness of such compensation.

This article is divided into several parts. In Section 2, a brief description of the detector and its principle of operation is given, followed by a description of the measurement methods, including the applied energies of radiation and the issue of the measurement accuracy using the internal temperature sensor, which is essential for further correction. The following section summarises the observed temperature effect and its distortion of energy measurements. Section 4 proposes and presents a comprehensive method for the correction of temperature distortion in the energy spectrum. Section 5 outlines the possibility of generalising the proposed model, including verification on a test sample. Finally, the penultimate Section 6 of the manuscript is devoted to testing over a wide range of temperatures, allowing a more expansive application field and testing the possibility of extrapolating the obtained model. The last section summarises and discusses the presented results and possible future research areas.

## 2. Material and Methods

As part of this research, several detectors were used and subjected to a series of experiments with radiation sources and different operating temperatures. The following sections provide a description of used instruments and methods.

### 2.1. Device Description

The Timepix3 is a hybrid pixel radiation detector as the successor to the widely used Timepix detector and the next addition to the Timepix/Medipix family of chips that were developed in the CERN laboratories. Compared to the previous model Timepix [29], TPX3 has better time resolution, adds functionalities, and improves on various electrotechnical specifications.

**Figure 1:**
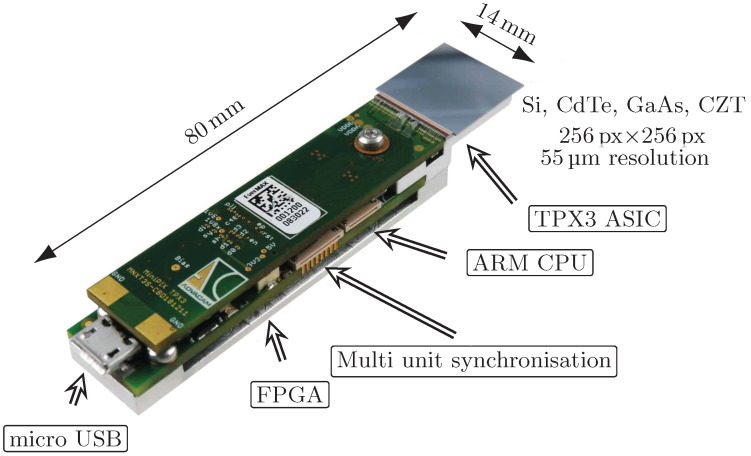
Illustration and description of the MiniPIX Timepix3 detector without a protective case.

It is a square-shaped detector with a size of 1.98 cm^2^ capable of measuring a broad range of ionising particles. There are (256 × 256) single pixels and a pitch of 55 μm, which makes it 65,536 individual pixels in total (see Figure 1). Each pixel is connected to the detector chip by a method called bump-bonding. At the same time, there is a set of integrated electronics for each pixel on the Application-Specific Integrated Circuit (ASIC) chip. It is mainly an analogue amplifier, a threshold discriminator, and an Analogue-to-Digital Converter (ADC). A great advantage of a pixel detector with an integrated Threshold (THL) discriminator is the possibility of using the THL to remove noise and thus operate the detector in near-noise-free mode. Unlike previous versions of Timepix detectors, which were able to read data only in the frame mode, when after the set acquisition time, the signal value is read from all pixels at once, TPX3 is also able to read data in the so-called data-driven mode. This mode does not rely on a uniform frequency and reads the signal from each individual pixel immediately after exceeding the Threshold of the given pixel. The data stream is thus continuous and with a much better time resolution. Since only hit pixels are read out, the total volume of data is significantly lower. The data stream is limited only by the dead time of individual pixels integrated electronics. Thus, its minimal value is, theoretically, 475 ns for low hit rates and matrix occupancy. When sensor occupancy is equal to or greater than 50%, the frame driven mode may be a better option than the data-driven mode in terms of readout time [30]. However, the data-driven mode will typically outperform the frame-driven mode for tracking individual particle tracks where no pileups are required. It is worth noting that the energy counter in the data-driven mode is limited to 1022 counts (approximately 500 keV per pixel), whereas in frame mode the integral energy counter has a range of up to 16,384 counts, making it more suitable for imaging applications with high photon or particle flux or measurements of highly ionising particles. The minimum threshold limit depends on the type and thickness of the used detection layer material and the internal noise of the ASIC chip. Typical detection layers can be Silicon (Si), Cadmium Telluride (CdTe), or Gallium Arsenide (GaAs) in various thicknesses [31,32]. For the Si detection layer, which was used in the presented work, the minimum threshold value is as low as 2 keV [33].

There are various measurement modes that suit different applications, allowing users to obtain information about various parameters of particles.

The Event Counting (EC) mode is a simple counting method that records the number of particles that hits a particular pixel. In this mode, the digital output of each pixel is monitored, and the count of rising edges (above the threshold) is recorded. This mode provides basic information about the particle flux and can be used for radiation mapping and dose rate monitoring.

The Time-of-Arrival (ToA) mode is a measurement mode that records the exact arrival time of incoming particles to each pixel. In this mode, the detector uses a time-stamping mechanism to record the time the particle crosses the threshold voltage level. ToA is a time indication of how much time has passed between the start of the measurement and the impact of the particle.

The Time-over-Threshold (ToT) mode involves the measurement of the duration of a signal generated by a particle exceeding a specified threshold voltage level. This mode implements an indirect energy measurement method based on the Wilkinson-type ADC, where the analogue signal from each pixel is continuously monitored. Whenever the signal exceeds the threshold voltage, a timer is triggered to start counting. The timer stops when the signal, which is being discharged through a constant current, falls below the threshold voltage. After calibration, the recorded time is proportional to the energy deposited by the particle in the pixel.

The Timepix3 detector can operate in one of three measurement modes (ToA, ToA & ToT, and EC & integralToT) [30], which combine above-mentioned principles and allow simultaneous measurement of some of the parameters. The frame-based readout mode allows measurement in the combination mode EC & integralToT, ToA & ToT and only ToA. This is different from the data-driven mode, which allows measurement in ToA & ToT, only ToA and only ToT.

The instruments used in this work were equipped with a readout interface called MiniPIX (see Figure 1). It is characterised by compact size, low power consumption, micro-USB connection, and the synchronisation of several units if required, and is equipped with Field Programmable Gate Array (FPGA) for fast data processing and detector handling. A set of five identical TPX3 detectors equipped with a 500 μm silicon sensor and a MiniPIX interface was created and will be further investigated in this paper.

### 2.2. Methods Descriptions

Two types of radiation sources were used to obtain data for radiation energy measurements. Radiation sources of the first type use the effect of X-ray fluorescence (XRF) to generate characteristic radiation. The XRF generates precise energy lines corresponding to the target material. The lines depend on the electron orbitals that are characteristic of atoms of a given element used as the target. The materials of the targets with corresponding emission lines used in this manuscript are listed in the first five rows of Table 1.

Due to the possibility of polychromatic background radiation from scattered radiation in a radiation-shielded box, the overall measured energy spectrum is complex. In addition to the mentioned specific energy lines, the spectrum includes X-rays produced by the X-ray fluorescence of the materials in the shielding box, mainly Lead, as well as photons that have undergone additional scattering either in the sensor or in the X-ray box itself, detected as Compton events. The measured spectrum also includes a continuous spectrum produced by the X-ray tube.

Two radionuclides Barium and Europium were used as a second type of radiation source. Their use provides sufficiently dense coverage of the energy spectrum up to 100 keV, which was chosen as the maximum reasonable value taking into account the detection efficiency of the sensors used. It should be noted that the detection efficiency of a 500 μm silicon sensor decreases sharply with increasing energy. It reaches about 50% at 20 keV, but is only 10% at 30 keV and continues to decrease with increasing incident energy. For this reason, the measurement time was extended with respect to the measurement energy of the peak under investigation and the efficiency of the sensor material. The radionuclide energy values that have been taken into account are listed in the last two rows of the table of sources used (Table 1).

**Figure 2:**
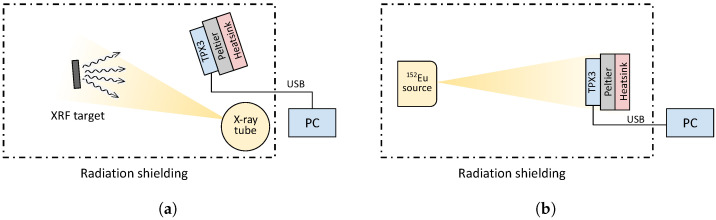
Schematic arrangement for measuring the energy spectrum of radiation. (**a**) X-ray fluorescence; (**b**) Radionuclides.

Each of these methods requires a different measurement arrangement. Therefore, two setups were prepared. To measure the energies generated by XRF, it was necessary to use an X-ray tube and a different geometrical arrangement compared to the application with radionuclides. Both setups use a holder for samples, and the whole arrangement was placed inside a radiation-shielded box. The schematic layouts are shown in Figure 2.

All detectors were placed and thermally coupled to a Peltier plate for their thermal stabilisation at each measurement phase. The energy measurements (ToT & ToA measurement in data-driven mode) of the individual samples were performed in temperature steps of 10 °C over a temperature range of 10 °C to 70 °C. Sufficient time for thermal stabilisation of the measurement setup was kept before each measurement started.

The evaluation methodology is based on the procedure presented in [28], where a combination of Gaussian $G_{a}\left(x\right)$ and complementary error function $E_{rfc}\left(x\right)$ is used (Equation (1)).

(1) $$G_{e}\left(E,A_{1},A_{2},\mu ,\sigma \right)=G_{a}\left(E,A_{1},\mu ,\sigma \right)+A_{2}\;\cdot \;E_{rfc}\left(\frac{E-\mu }{\sigma \sqrt{2}}\right)$$

The function $G_{a}\left(E,A_{1},\mu ,\sigma \right)$ stands for the Gaussian

(2) $$G_{a}\left(E,A_{1},\mu ,\sigma \right)=A_{1}\;\cdot \;e^{\frac{-{\left(E-\mu \right)}^{2}}{2\sigma^{2}}}$$

and complementary error function is defined as

(3) $$E_{rfc}\left(x\right)=1-\frac{2}{\sqrt{\pi }}\int_{0}^{x}e^{-t^{2}}dt.$$

**Figure 3:**
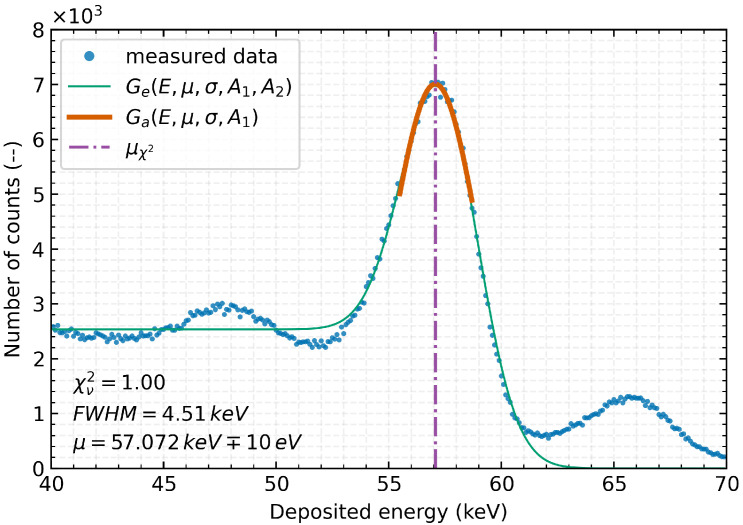
Part of the measured energy spectrum of the radiation produced by the X-ray fluorescence of a Tantalum target with a 500 μm Si Timepix3 detector without temperature stabilisation. Measured data (blue markers) together with the $G_{e}\left(E,A_{1},A_{2},\mu ,\sigma \right)$ function (combination of Gaussian and complementary error function—green line) and with the result of the final fit of the $G_{a}\left(E,A_{1},\mu ,\sigma \right)$ function (orange line) to the Ta-$K_{\alpha 1}$ line. The resulting position of the $\mu_{\chi^{2}}$ of the evaluated peaks determined by $\chi^{2}$ minimisation is 57.072 keV (purple dashed dotted line). Considering the statistically significant number of observed events, the maximum size of statistical uncertainties was determined to be <80 counts in the range shown, the individual error bars are not plotted to the measured points for clarity.

The following notation is used for the energy (*E*), the intensity of the error function ($A_{2}$) or intensity of the spectral peak ($A_{1}$) and its mean energy (μ) as well as the energy noise (σ). Figure 3 shows an example of the evaluation procedure where function (Equation (1)) (green line) is applied over the range of measured data (blue markers) to provide an initial estimation of the input parameters for the accurate determination of the measured energy peak parameter.

The obtained values can then be used to automatically determine a suitable evaluation range and input parameters to accurately fit the Gaussian function (Equation (2)) of the energy peak near its maximum intensity, see orange line in Figure 3.

Goodness of fit and its quality are assessed using Pearson’s chi-square ($\chi^{2}$) test (Equation (4)), respectively, reduced chi-square ($\chi_{\nu }^{2}$) statistic (Equation (5)). It is possible to fine-tune the position of the mean ($\mu_{\chi^{2}}$) of the examined energy peak according to the distribution of $\chi^{2}\left(\mu \right)$, particularly the position of its minimum, under the conditions of the fit acceptability $\chi_{\nu }^{2}\approx 1$.

(4) $$\chi^{2}=\sum_{i}\frac{{\left(M_{i}-F_{i}\right)}^{2}}{\sigma_{i}^{2}}$$

(5) $$\chi_{\nu }^{2}=\frac{\chi^{2}}{\nu }$$

The variable *i* defines the evaluation interval, which in this case corresponds to the range of the Gaussian fit, $M_{i}$ represents the number of measured counts in the *i*-th energy bin, $F_{i}$ is the value of the expected flux based on the estimated Gaussian fit, and $\sigma_{i}^{2}$ represents the variance of the individual counts. The $\chi_{\nu }^{2}$ is defined as the proportion of $\chi^{2}$ and the degrees of freedom (ν), where $\nu =\left|i\right|-3$ with $\left|i\right|$ as the cardinality of the evaluated interval *i*.

According to the procedure described, the position of the energy peak (see purple dashed-dotted line in Figure 3) at 57.072 keV can be determined with an uncertainty of 10 eV from the given data of the Ta-$K_{\alpha 1}$ line measurement, and the Full Width at Half Maximum (FWHM) is determined to 4.51 keV.

The variation of these parameters, in particular, the drift of the peak mean (absolute measurement accuracy, relative error of measurement) and its width (relative energy resolution), are further compared and evaluated. The reference values for absolute measurement accuracy and relative error of measurement are obtained from measurements at 20 °C, the temperature at which the manufacturer calibrates the detectors.

### 2.3. Internal Temperature Sensor

The stability of the measured energy spectrum is crucial and is achieved by maintaining a constant or known temperature. The internal thermometer on the chip should provide sufficient thermal accuracy, and the calibration of this thermometer is necessary for the proper energy shift compensation. This chapter discusses the verification of the linearity of the internal thermometer using an external reference PT1000 thermometer. The several selected temperatures in the range of 10 °C to 70 °C were chosen to be relevant to the operational temperature range of the TPX3 detector.

Timepix3 detector has two places on-board to measure temperature. The first and most crucial is in the TPX3 ASIC chip, and the second is an internal thermometer of the processing microcontroller. Both integrated circuits were thermally coupled to the Peltier plate and stabilised on the desired temperature in the range from 10 °C to 70 °C and controlled by an external device until the measured temperature was stable on all three thermometers. It takes about 10 min to stabilise the plate at each point.

During the thermal cycling process for spectral characterisation, temperature data was collected from all detectors. The results of the investigation indicated that all thermometers exhibited a linear progression in their readings, with a slight offset compared to the preset temperature. Therefore, the relative error of temperature measurement was noted to be between 1% and 40% depending on the specific device and the measured temperature.

A linear regression calculation was performed for the ASIC chip, resulting in the regression between 0.94 and 1.02 for all five measured detectors, as stated in the row slope in Table 2, which follows the preset temperature linearly but with a constant offset. The constant offset was estimated to range from −6.06 °C to 0.8 °C and the coefficient of determination R-squared (R^2^) was found to be nearly 1 for all measured detectors, which indicates that linear regression is a suitable fit for the internal thermometer.

In case the estimated value of slope differs from 1 in the linear regression, the deviation from the set and measured temperature will vary if not corrected. Based on the coefficients listed in Table 2, it is possible to determine the correction function for the internal thermometers. Given the linear dependence, it is sufficient to measure the temperature at two points, such as room temperature and 50 °C for example, to determine the linear function. This step is crucial in obtaining accurate information about the detector temperature during measurement because, as shown further in the article, the detector’s thermal variation significantly affects the resulting energy spectrum and information about its value is critical for the correction.

## 3. Temperature Effects

**Figure 4:**
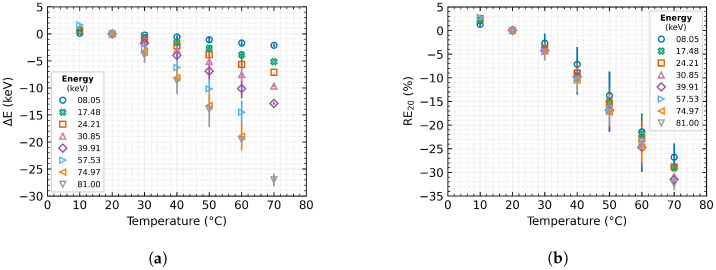
Influence of temperature change of the MiniPIX Timepix3 detectors equipped with 500 μm Si sensor on the obtained energetic spectrum measured by Time-over-Threshold in data-driven mode. The plotted data represent the mean values over the tested detectors, and the error bars indicate their minimum and maximum value: (**a**) Absolute measurement accuracy; (**b**) Relative error of measurement.

Based on the measured and processed data from all examined detectors, radiation sources, and temperature stages, the temperature dependence of the Timepix3 detectors [28] was verified. The results in Figure 4 represent the significant temperature dependence of the radiation energy measurements in the Time-over-Threshold mode of the detectors.

The results show that the energy spectrum drifts with the temperature changes. With increasing temperature, the energy spectrum nonlinearly shifts towards lower energies, this offset is more significant with increasing incident energy. Already with a 10 °C change of the detector temperature, a relative measurement error of about 4% occurs. A further change in temperature from the reference point at 20 °C, e.g., due to the influence of the environment or the detector self-heating, may cause a displacement of the energy spectrum of up to tens of kiloelectronvolts. This drift causes an error of more than 30%, in the case of heating the detector to 70 °C.

Table 3 shows the resulting values for selected radiation sources and detector temperatures from the tested interval.

## 4. Compensation Method

As was written in the recently published paper, a simplified linear compensation [28] can be used for the initial compensation of the detector’s temperature drift. This compensation method is effective for the constant temperature difference of the detector during measurement from the calibration temperature. The linear compensation method indeed finds its use; however, if the detector is permanently exposed to different but constant conditions, performing a complete recalibration of the device under these conditions is preferable. Nevertheless, if the condition varies frequently, this approach is not appropriate. A new method of complex temperature compensation is proposed, which is based on the analysis of the collected data, the results, and the observed dependencies. This method utilises a temperature distortion model applicable to a continual change not only of the detector temperature but also of the incident radiation energy.

**Figure 5:**
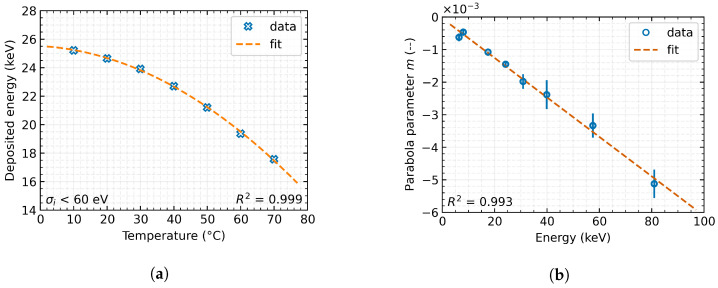
Model of temperature dependence distortion of measurement accuracy of radiation energy spectrum (Time-over-Threshold in Data-driven mode) with Timepix3 detector equipped with 500 μm Si sensor. The error bars indicate the inaccuracy of a given parameter obtained by the fitting function: (**a**) Temperature dependence; (**b**) Energy dependence.

The examination of the measured data and subsequent processing (Figure 4) suggests a definable temperature dependence of the energy spectrum drift. Further processing allows us to describe the dependence of the measured energy on temperature (Figure 5a) and thus to establish Equation (6).

(6) $$E_{meas}=mT^{2}+nT+o$$

where $E_{meas}$ is measured energy by the detector, *T* is detector temperature during measurement and *m*, *n*, *o* are function parameters.

However, the parameters of this dependency are highly variable concerning the energy of the incident radiation. Therefore, it was necessary to define the interdependence of this function on the radiation energy. Gradually, it became apparent that the individual parameters of the Equation (6) have a linear dependence with regard to the radiation energy (Figure 5b) and, therefore, can be described by linear regression, see Equation (7).

(7) $$x=x_{1}E+x_{2}$$

where *x* represents one of the parameters *m*, *n*, *o* in Equation (6), *E* is the energy of radiation, and $x_{1}$, $x_{2}$ are parameters of the dependence.

The mutual substitution of these two functions yields a model of the temperature distortion of the measurement accuracy of the energy spectrum with the TPX3 detector. The expression *E* leads to a function (Equation (8)) that contains the constants *m*, *n*, and *o* obtained from the model and the two variables $E_{meas}$ as the measured energy and *T* as the temperature of the measurement.

(8) $$E=\frac{E_{meas}-m_{2}T^{2}-n_{2}T-o_{2}}{m_{1}T^{2}+n_{1}T+o_{1}}$$

Its application to the measured data yields the real energy of the incident radiation after correction for temperature distortion.

**Figure 6:**
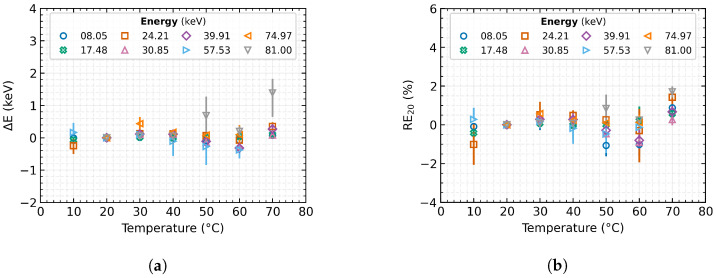
The change in absolute measurement accuracy and relative measurement error of the tested sources after applying the proposed correction. The plotted data represent the mean values over the tested detectors, and the error bars indicate their minimum and maximum value: (**a**) Absolute measurement accuracy; (**b**) Relative error of measurement.

The evolution of absolute measurement accuracy and relative measurement error over the complete, tested range after applying the presented correction method is shown in Figure 6. The results are compiled over all tested detectors. A simple comparison with Figure 4 demonstrates the significant minimisation of the temperature dependence, the compensation of the thermal drift in the energy spectrum of the radiation, and the improvement of the obtained results. The plot shows that the absolute shift of the measured energy has been reduced by applying the correction from almost −30 keV to ±0.5 keV over the entire tested energy and temperature range. The relative error of the measurements has thus also dropped from the original −30% (at 70 °C) to 1.5% thus staying within the ±1.5% tolerance interval over the whole range. For a more accurate representation, Table 4 gives specific values for the same selected radiation source types and detector temperatures as in Table 3.

## 5. Generalisation

As part of the potential expansion of the presented compensation method for a wide range of applications without the necessity to characterise individual detectors, the possibility of parameter generalisation was also tested. Based on the tested detectors’ data, an average temperature dependence model was established and the necessary general parameters for the compensation were determined (see Table 5). One detector was removed from the modelling set for generalisation and has been used for final verification and applicability comparison.

**Figure 7:**
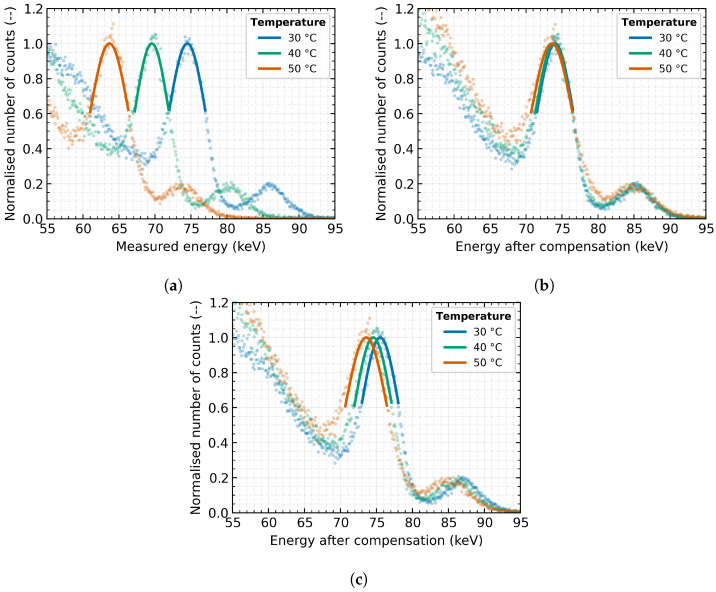
The comparison of the energy spectrum obtained by X-ray fluorescence with a Lead target and different compensation for thermal distortion. The measurements were performed with the detector *E* at three different sensor temperatures (30 °C, 40 °C and 50 °C): (**a**) Without compensation; (**b**) Individual compensation model; (**c**) Generalised compensation model.

The characteristic X-ray fluorescence of the Lead target (energy 74.97 keV) was measured several times with detector *E* outside the modelling set at detector temperatures of 30 °C, 40 °C and 50 °C. The resulting measured spectrum after normalisation for comparison is shown in Figure 7a. As can be seen, the temperature effect significantly distorts the accuracy of the energy measurement by 19%.

Applying the above-proposed compensation method with the parameters obtained from the previous characterisation of the detector *E*, the energy spectrum shown in Figure 7b can be produced. It can be seen from the figure that the effect of temperature distortion on the absolute accuracy of the measurement was nearly minimised to the relative error of measurement of less than 0.7%. At the same time, there is a clear trend of increasing energy peak width, common to all measurements and methods, thus deteriorating the detector’s energy resolution for high temperatures. This is due to the increasing thermal noise in the device.

Suppose the generalised parameters listed in Table 5 are used in the same correction method instead of using the individual parameters for a given detector. In that case, the spectra shown in Figure 7c are obtained. It should be noted that a more statistically significant number of detectors would need to be tested and characterised to produce a robust, generally valid, generalised model.

It is noticeable that the generalised compensation does not provide as satisfactory results as in the prior case. This result was expected because when using generalised parameters for the compensation model, there are always compromises regarding the specific parameters of the detectors included in the training set. Therefore, after applying the generalised parameters, the output cannot be as accurate as in the case of compensation parameters determined for a specific detector. Nevertheless, it is shown that the proposed compensation method can be successfully applied to correct the energy drift even with generalised parameters based on several other detectors. The resulting relative error of the measurements does not exceed 1.5%, for the particular detector types. The detailed comparison of the fundamental parameters for several detector temperatures is given in Table 6 for the cases: raw measured, compensation based on parameters for specific detector and after compensation of thermal distortion based on generalised parameters.

## 6. Validation and Extrapolation over a Wide Temperature Range

The validation of a model over a wide temperature range is a particularly important process that can significantly increase the applicability and versatility of a given model in engineering and scientific applications, such as aerospace, automotive, or outdoor environmental research. This reduces the risk of failure in safety-critical or mission-critical applications.

The extrapolation of measured data to temperatures below 0 °C can effectively reduce the need for complex and costly measurements in extreme temperature conditions, while still providing valuable insight into system behaviour. This approach can lead to more efficient and cost-effective product or system development/application without the need for a number of physical tests and additional calibrations. The utility of such extrapolation has been demonstrated in this paper, where the model has been validated over a temperature range including temperatures down to −40 °C.

**Figure 8:**
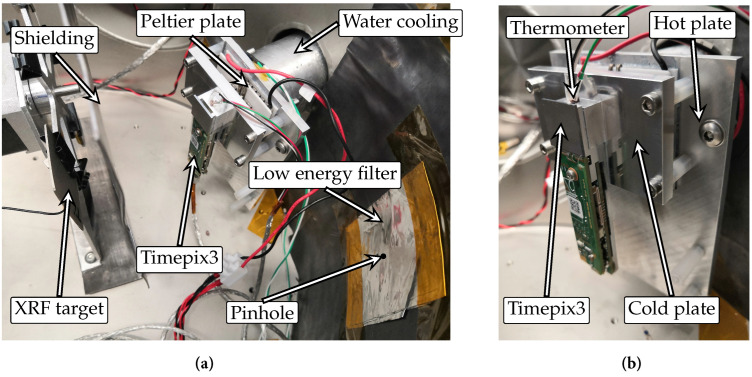
Timepix3 detector arrangement for thermal testing of verification and extrapolation of the compensation model in the vacuum chamber: (**a**) arrangement configuration for X-ray fluorescence measurement; (**b**) details of the detector placement on the Peltier module with feedback thermometer.

A detector MiniPIX TPX3 with a 1000 μm thick Silicon sensor, beyond the previously tested collection, was used to verify the proposed model and the extrapolation. It was operated at temperatures between −40 °C to 60 °C, requiring careful control of the test environment to prevent condensation and frosting on the detector because temperatures were deep below the dew point. Consequently, the device under test could have been short-circuited and irreversibly damaged. To mitigate this risk, the detector was placed in a vacuum chamber and thermally coupled and stabilised using a three-stage Peltier element (Figure 8). Waste heat was dissipated through a water cooling system outside the vacuum chamber. The temperature was stabilised for at least 10 min before each measurement and controlled with a feedback thermometer placed below the Timepix3 chip. An automatic carousel with multiple targets for generating XRF radiation at various energies was also placed inside the chamber.

Due to limitations in the vacuum system and safety measures, it was possible to generate characteristic fluorescence radiation with energies up to 30 keV. Considering these restrictions and with respect to the material and thickness of the detection layer, the following XRF targets were selected: Zircon (Zr) at 15.77 keV, Cadmium (Cd) at 23.11 keV and Tin (Sn) at 25.27 keV. Additional to the X-ray fluorescence targets, an Americium (^241^Am) radionuclide with a spectral line at 59.54 keV has been selected for optimal results.

**Figure 9:**
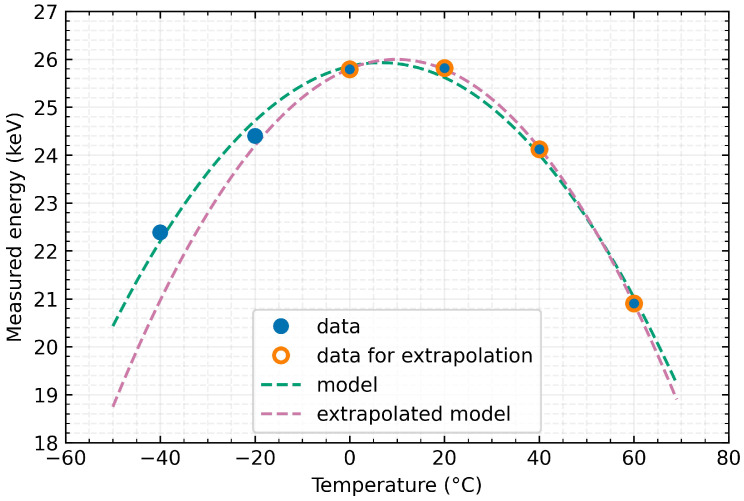
Model of temperature compensation of energy measurement (X-ray fluorescence with Tin target and detector in Time-over-Threshold mode of measurement and data-driven readout mode) distortion using Timepix3 detector with 1000 μm Silicon sensor in the temperature range from −40 °C to 60 °C. Extrapolated model based on data measured in temperatures above 0 °C is shown as well.

**Figure 10:**
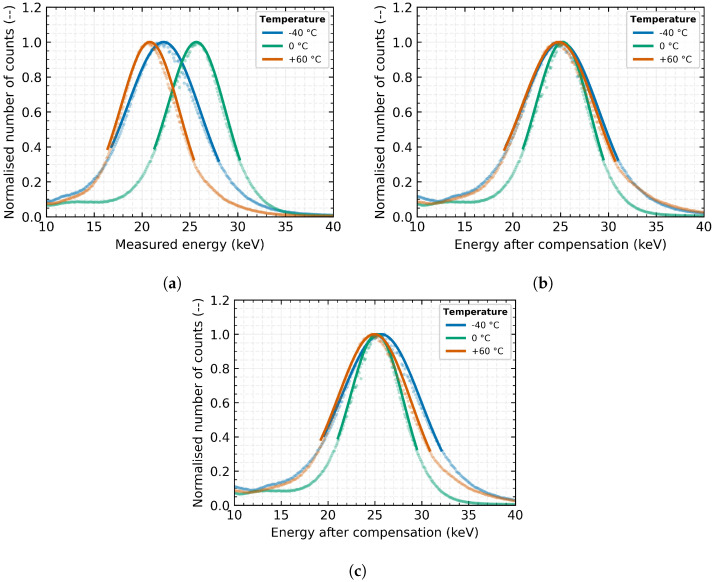
The comparison of the energy spectrum obtained by X-ray fluorescence with a Tin target and different compensation for thermal distortion. The measurement results of a detector with a 1000 μm Si sensor placed in a vacuum for different sensor temperatures (−40 °C, 0 °C and 60 °C) are shown: (**a**) Without compensation; (**b**) Individual compensation model; (**c**) Extrapolated compensation model.

From Figure 9, it is clear that the described and presented model for compensating temperature distortion of the measured energy spectrum of the incident radiation and, thereby, the increasing error of energy measurement is not only effective for temperatures above 0 °C but has also been validated at temperatures below freezing point. The results of experiments verified that the superimposed parabolic model effectively reduces the relative measurement error at −40 °C from −11% to approximately 0.5% (for XRF energy of Sn; 25.27 keV) as can be clearly seen in the comparison of Figure 10a,b.

The extrapolation of the above-mentioned model was tested with the same detector. A temperature compensation model was developed based on the data points that were above the freezing point (depicted as orange in Figure 9). Figure 10 reveals that as the temperature decreased and moved away from the reference points, the extrapolated model showed an increased discrepancy from the model constructed using the entire dataset. However, even this extrapolated model provides a considerable improvement in the relative accuracy of the measurement for temperatures below 0 °C (as seen in Figure 10c). At a temperature of −40 °C, it reduces the error from the original by more than −11% to approximately 2% (as shown in Table 7). This compensation may be sufficient for some applications, and extrapolation would simplify and speed up the compression process when it is not necessary or practical to create suitable cooling conditions and it is difficult to undercool the detector for parameterisation.

## 7. Conclusions

This paper presents a solution to the problem of energy spectrum distortion due to temperature fluctuations in the Timepix3 detector with MiniPIX readout interface and Silicon sensor with a thickness of 500 μm and 1000 μm. The study’s results demonstrate that a relative measurement error of up to 35% can occur in the tested temperature range from 10 °C to 70 °C. The study was carried out using different radiation sources (X-ray fluorescence and natural sources) in the field of up to 100 keV to investigate the detector’s behaviour.

The first part of the paper also emphasises the issues of the potential usage of an internal thermometer built into the ASIC chip, which would significantly impact the measurement and compensation capabilities.

The authors propose a complex compensation method, which has been demonstrated to minimise the measurement error to no more than 1.5% for all energies over the entire temperature range tested. The results show that the proposed compensation method and models significantly improve the accuracy of energy measurements in Time-over-Threshold mode.

The possibility of creating a general model for temperature distortion compensation was also discussed. It would require testing a statistically significant number of detectors. Nevertheless, the results of the study indicate that even when applied to a small set of five, detectors, the possibility of generalisation appears to be realistic. After applying the generalised model to a detector not included in the learning set, the relative error of energy measurement in the X-ray fluorescence spectrum of Lead (74.97 keV) was found to be reduced from the original 15% to less than 1.5% at a detector temperature of 50 °C.

The compensation method was further tested in temperatures up to −40 °C using a vacuum chamber and was also found to be valid in this range. The extrapolated model, based on measurements where the temperature did not drop below 0 °C, was verified for the correction of energy spectra measured when the detector temperature was below 0 °C. It was found that although the efficiency of the compensation of the measurement error of the incident energy is degraded, the extrapolated model can be used. During the tests with XRF of the Tin target (25.27 keV) and using at a detector temperature of −40 °C, the relative measurement error was reduced from the original 11.4% to 2.1% with extrapolated model (0.5% in the case of the individual model based on all data points).

The presented results, methods, and models allow the use of the TPX3 detector to be extended to a wide range of applications where cooling or temperature stabilisation of the detector is not possible or economically viable. The compensation method provides a valuable solution to a common problem in space radiation measurement and has the potential to have a significant impact on various applications.

In conclusion, the authors have successfully demonstrated a solution to the problem of energy spectrum distortion due to temperature fluctuations in the Timepix3 detector with MiniPIX readout interface and Si sensor with a thickness of 500 μm and 1000 μm. The proposed compensation method has been shown to significantly improve the accuracy of energy measurements, and the model’s validity has been verified in both parts of the temperature range, below and above 0 °C. The findings of the study have the potential to impact a wide range of applications and industries, increase the application potential of this detector type in other fields and decrease workforce demands (series of calibrations) and power sources (thermal stabilisation/cooling).

## Figures and Tables

**Table 1:** List of radiation sources and target materials with their characteristic energies.

Symbol	Element/Radiation Source	Energy
Cu	Copper	8.046 keV
Mo	Molybdenum	17.480 keV
In	Indium	24.209 keV
Ta	Tantalum	57.532 keV
Pb	Lead	74.969 keV
^152^Eu	Europium	39.910 keV
^133^Ba	Barium	30.973 keV
		80.998 keV

**Table 2:** Measured linearity, offset and coefficient of determination (R^2^) of the temperature dependence of five Timepix3 detectors on preset temperature by an external device.

	Chip A	Chip B	Chip C	Chip D	Chip E
**Slope**	1.01	1.00	0.94	0.99	1.02
**Offset**	−4.13	−0.76	0.80	−3.10	−6.06
**R^2^**	0.9999	0.9999	0.9997	1.0000	0.9997

**Table 3:** Temperature influence on radiation energy measurement with 500 μm silicon MiniPIX Timepix3 detectors in Time-over-Threshold mode. Where ΔE is the absolute measurement accuracy, RE is the relative error of measurement, and Res. is the relative energy resolution.

Energy	17.48 keV			24.21 keV			57.53 keV		
**Param.**	**ΔE (keV)**	**RE (%)**	**Res. (%)**	**ΔE (keV)**	**RE (%)**	**Res. (%)**	**ΔE (keV)**	**RE (%)**	**Res. (%)**
**10 °C**	0.37	2.11	16.88	0.62	2.52	13.24	1.64	2.79	8.70
**30 °C**	−0.68	−3.83	17.02	−0.92	−3.73	12.72	−2.67	−4.50	6.48
**60 °C**	−3.89	−21.95	18.66	−5.66	−22.87	15.20	−14.51	−24.49	8.85

**Table 4:** Temperature influence on radiation energy measurement with 500 μm silicon MiniPIX Timepix3 detectors in Time-over-Threshold mode after application of the proposed individual correction method. Where ΔE is the absolute measurement accuracy, RE is the relative error of measurement, and Res. is the relative energy resolution.

Energy	17.48 keV			24.21 keV			57.53 keV		
**Param.**	**ΔE (keV)**	**RE (%)**	**Res. (%)**	**ΔE (keV)**	**RE (%)**	**Res. (%)**	**ΔE (keV)**	**RE (%)**	**Res. (%)**
**10 °C**	−0.07	−0.42	14.99	−0.25	−1.02	14.01	−0.20	−0.35	8.67
**10 °C**	0.01	0.08	16.21	0.13	0.52	11.64	0.07	0.13	6.67
**60 °C**	0.04	0.21	22.56	−0.07	−0.31	18.80	−0.56	−0.97	12.52

**Table 5:** Generalised parameters to compensate for temperature distortion in energy spectrum measurement accuracy using Timepix3 detectors with 500 μm Silicon sensor.

Parameter	$X_{1}$	$X_{2}$
**m**	−5.66427 × 10^−5^	−1.27101 × 10^−4^
**n**	−1.66014 × 10^−3^	2.76756 × 10^−2^
**o**	0.10911 × 10^1^	−0.10019 × 10^1^

**Table 6:** Comparison of the energy spectrum parameters obtained by X-ray fluorescence with a Lead target and different compensation for thermal distortion. Measurements were performed with detector *E* (which is outside the modelling set) at three different sensor temperatures (30 °C, 40 °C and 50 °C). Symbol ΔE represents the absolute measurement accuracy, RE is the relative error of measurement, and Res. is the relative energy resolution.

	Measured			Individual			Generalised		
**Param.**	**ΔE (keV)**	**RE (%)**	**Res. (%)**	**ΔE (keV)**	**RE (%)**	**Res. (%)**	**ΔE (keV)**	**RE (%)**	**Res. (%)**
**30 °C**	−3.45	−4.38	5.93	−0.41	−0.55	6.61	1.10	1.46	6.73
**40 °C**	−8.49	−10.79	6.06	−0.43	−0.57	6.78	0.27	0.37	6.87
**50 °C**	−14.87	−18.89	8.20	−0.52	−0.70	8.18	−0.56	−0.74	8.30

**Table 7:** Comparison of the energy spectrum parameters obtained by X-ray fluorescence with a Tin target and different compensation for thermal distortion. The measurement results of a detector with a 1000 μm Silicon sensor placed in a vacuum for different sensor temperatures (−40 °C, 0 °C and 60 °C) are listed. Where ΔE is the absolute measurement accuracy, RE is the relative error of measurement, and Res. is the relative energy resolution.

	Measured			Individual			Extrapolated		
**Param.**	**ΔE (keV)**	**RE (%)**	**Res. (%)**	**ΔE (keV)**	**RE (%)**	**Res. (%)**	**ΔE (keV)**	**RE (%)**	**Res. (%)**
**−40 °C**	−2.88	−11.41	35.17	−0.13	−0.5	36.95	0.54	2.13	39.43
**0 °C**	0.31	2.03	27.95	0.04	0.14	26.38	0.02	0.09	26.48
**60 °C**	−4.36	17.27	28.29	−0.35	−1.39	36.09	−0.21	−0.84	36.26

## Data Availability

The data presented in this study are available from the corresponding author on request.
